# How a faecal immunochemical test screening programme changes annual colorectal cancer incidence rates: an Italian intention-to-screen study

**DOI:** 10.1038/s41416-022-01813-7

**Published:** 2022-04-20

**Authors:** Lauro Bucchi, Silvia Mancini, Flavia Baldacchini, Alessandra Ravaioli, Orietta Giuliani, Rosa Vattiato, Federica Zamagni, Paolo Giorgi Rossi, Cinzia Campari, Debora Canuti, Enza Di Felice, Priscilla Sassoli de Bianchi, Stefano Ferretti, Nicoletta Bertozzi, Annibale Biggeri, Fabio Falcini, the Emilia-Romagna Region Workgroup for Colorectal Screening Evaluation, Enza Di Felice, Alba Carola Finarelli, Patrizia Landi, Carlo Naldoni, Priscilla Sassoli de Bianchi, Americo Colamartini, Elisabetta Borciani, Fabio Fornari, Giorgio Gatti, Francesca Pennini, Pietro Seghini, Cristian Dalla Fiora, Claudio Fattibene, Fabio Maradini, Maria Michiara, Paolo Orsi, Corrado Zurlini, Lucia Mangone, Luisa Paterlini, Romano Sassatelli, Giuliano Carrozzi, Rossella Corradini, Federica Rossi, Paolo Trande, Simona Viani, Carmen Bazzani, Franco Bazzoli, Vincenzo Cennamo, Chiara Giansante, Giovanna Gualandi, Marilena Manfredi, Francesca Mezzetti, Adriana Pasquini, Licia Caprara, Margherita De Lillo, Roberto Nannini, Maria Cristina Carpanelli, Aldo De Togni, Vincenzo Matarese, Caterina Palmonari, Daniela Pasquali, Giorgio Zoli, Serena Dal Re, Chiara Petrini, Monica Serafini, Benedetta Vitali, Mara Gallinucci, Claudia Imolesi, Mauro Palazzi, Enrico Ricci, Mirna Severi, Coralba Casale, Mauro Giovanardi, Daniele Trombetti

**Affiliations:** 1 Romagna Cancer Registry, Romagna Cancer Institute, IRCCS Istituto Romagnolo per lo Studio dei Tumori (IRST) “Dino Amadori”, Meldola, Forlì, Italy; 2 Epidemiology Unit, Azienda Unità Sanitaria Locale—IRCCS di Reggio Emilia, Reggio Emilia, Italy; 3 Cancer Screening Unit, Azienda Unità Sanitaria Locale—IRCCS di Reggio Emilia, Reggio Emilia, Italy; 4 Cancer Screening Unit, Local Health Authority, Rimini, Italy; 5 Department of Health, Regional Administration, Emilia-Romagna Region, Bologna, Italy; 6 University of Ferrara and Local Health Authority, Ferrara, Italy; 7 Unit of Biostatistics, Epidemiology and Public Health, Department of Cardiac, Thoracic, Vascular Sciences and Public Health, University of Padua, Padua, Italy; 8 Cancer Prevention Unit, Local Health Authority, Forlì, Italy; 9 Local Health Authority, Piacenza, Italy; 10 Local Health Authority, Parma, Italy; 11 Local Health Authority, Modena, Italy; 12 Local Health Authority, Bologna, Italy; 13 Local Health Authority, Imola, Italy; 14 Local Health Authority, Ferrara, Italy; 15 Local Health Authority, Ravenna, Italy; 16 Local Health Authority, Cesena, Italy; 17 Local Health Authority, Rimini, Italy

**Keywords:** Preventive medicine, Cancer screening

## Abstract

**Background:**

This study aimed to evaluate the effectiveness of a biennial faecal immunochemical test (FIT) screening programme in reducing annual colorectal cancer (CRC) incidence in its dynamic target population.

**Methods:**

The target population included over 1,000,000 persons aged 50–69 living in a region of northern Italy. The average annual response rate to invitation was 51.4%. Each observed annual age-standardised (Europe) rate per 100,000 persons between 2005, the year of introduction of the programme, and 2016 was compared with each expected annual rate as estimated with age-period-cohort (men) and age-period (women) models.

**Results:**

For both sexes, the rates observed in 1997–2004 and those expected in 2005–2016 were stable. Observed rates increased in 2005, peaked in 2006 (the first full year of screening), dropped significantly below the expected level in 2009, and continued to decrease until 2013 (the eighth full year), after which no further significant changes occurred. In the pooled years 2013–2016, the observed incidence rate per 100,000 persons was 102.2 [95% CI: 97.4, 107.1] for men, 75.6 [95% CI: 71.6, 79.7] for women and 88.4 [95% CI: 85.3, 91.5] for both sexes combined, with an observed:expected incidence rate ratio of 0.68 [95% CI: 0.65, 0.71], 0.79 [95% CI: 0.76, 0.82] and 0.72 [95% CI: 0.66, 0.81], respectively.

**Discussion:**

The study provided multiple consistent proofs of a causal relationship between the introduction of screening and a stable 28% decrease in annual CRC incidence after eight years.

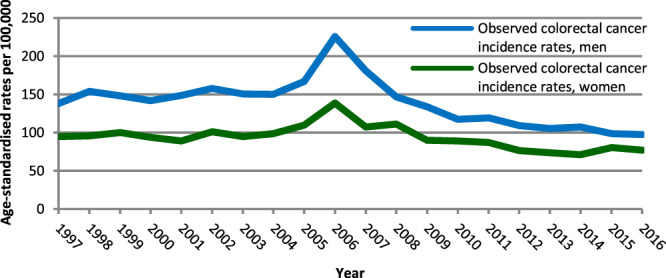

## Introduction

The adenoma-carcinoma sequence is the pathway by which most colorectal cancers (CRCs) arise and provides the strongest rationale for screening for the disease [1]. Preventing the progression of CRC and reducing its incidence contributes to mortality reduction and is the key factor for the cost-effectiveness of screening [2, 3]. In the light of the projected constant increase in expenditure for CRC treatment [3], an impact on incidence will become an increasingly valuable effect of screening, especially of public health screening programmes.

Randomised controlled trials and population-based case-control studies have shown that both sigmoidoscopy and colonoscopy screening, followed by polypectomy if indicated, reduce the risk of CRC [4–9]. As regards faecal occult blood test (FOBT) screening, with the colonoscopic evaluation of subjects who test positive, the evidence for an impact on CRC incidence relies solely on the results of the Minnesota trial, in which the guaiac FOBT was used [10]. Other guaiac-based FOBT screening trials have demonstrated a mortality benefit but not a decrease in the risk of disease [11–14].

Currently, however, major guidelines worldwide recommend faecal immunochemical tests (FITs) in preference to FOBTs [15, 16]. According to the US Multi-Society Task Force on CRC, for example, the annual FIT is one of the two cornerstones of CRC screening—the other being colonoscopy every 10 years [17]. FITs are analytically more sensitive for CRC and advanced adenoma than FOBTs [18–20]. Consequently, newer tests are expected to have a greater effect on CRC incidence [21].

In fact, experimental data to confirm this anticipation are lacking. There are no controlled trials demonstrating that FITs are superior to FOBTs or no screening and, based on the knowledge acquired thus far, it would be no longer ethical to randomise people to no intervention. To bridge this knowledge gap, computer simulation modelling has been increasingly used. Many such studies have supported the effectiveness of FIT screening in reducing the risk of CRC [2, 3, 22].

The rationale for the present study was the assumption that further evidence for the impact of FIT screening can be obtained with well-designed and well-powered observational studies in the context of ongoing public health screening programmes. This type of research has not yet been conducted extensively, but this is because the implementation of these activities is, in general, of recent date. Two previous small studies have shown a significant decrease in CRC incidence by comparing screening participants with non-participants (per-protocol analysis) [23] and the whole invited population with a historical control population (intention-to-screen analysis) [24]. A mid-term cohort study has found a 25% decrease in CRC rates after the implementation of an organised approach to screening in a community-based health care delivery system, but colonoscopy was used as a screening test in almost an equal proportion of people as FIT [25].

More consistent evidence for the impact of FIT screening on CRC incidence has been reported by a recently published cohort study comparing attenders with non-attenders (per-protocol analysis) to a screening programme that has been ongoing since 2005 in the Emilia-Romagna Region (northern Italy) for a dynamic resident population of over 1,000,000 people aged 50–69 years [26]. Attendance, as compared with non-attendance, was associated with a self-selection-adjusted reduction in CRC rates of 33% among men and 21% among women. The study reported here evaluates the effects of the same programme using an intention-to-screen design. Its purpose was to assess the changes in annual CRC incidence rates in the whole target population over a time span of 20 years (1997–2016) including 11 full years of screening.

## Methods

### Setting

The programme is run by 11 health care district screening units according to a standard protocol developed at the Department of Health of the Regional Administration. Every two years, subjects in the target age range are sent a personal letter inviting them to perform a one-sample FIT without dietary restrictions (OC-Sensor, Eiken Chemical Co., Tokyo, Japan). As reported elsewhere [27], the vast majority of FITs are distributed by public pharmacies and primary care centres. The FITs are analysed in the laboratories of public hospitals under strict internal and external quality assurance procedures. The haemoglobin concentration cut-off is ≥20 μg Hb/g faeces.

Negative FIT results are notified by mail. Subjects who test positive are contacted in person by telephone and invited to attend the screening centres, where they are referred for a complete colonoscopy under sedation. Colonoscopies are performed in public hospitals by qualified gastroenterologists. In the case of an incomplete colonoscopy, a computed tomographic colonography is performed. Subjects with screen-detected neoplasms are referred for endoscopic or surgical treatment. Subsequent follow-up is delivered in the clinical setting according to a standard protocol. Subjects with positive FIT results and negative colonoscopy are reinvited to FIT screening five years later. A dedicated colonoscopic screening programme for subjects with a family history of CRC, nested in the FIT screening programme, has been discontinued in 2013 [28].

The year 2005 was only partially covered by the programme. The first round was completed by 2007, in approximately two years. Supplementary Table S1 shows a set of average annual performance measures of the screening process between 2005 and 2016, calculated for three arbitrary 4-year periods. The major quality indicators established by the Italian Group for Colorectal Cancer Screening are included [29]. The 12-year average annual proportion of subjects responding to the invitation was 51.4%. Previous studies have analysed the results of the screening programme among compliant participants [30], and the proportional incidence of interval CRC among subjects testing negative on FIT [31].

### Objectives

In this article, we report an intention-to-screen analysis aimed to: (i) identify significant changes in CRC incidence in the dynamic target population and assess their temporal correlation with the introduction of the screening programme; (ii) estimate the annual incidence rates that would be expected in the absence of screening; (iii) compare the observed annual incidence rates with those expected; (iv) estimate the annual and cumulative screening-attributable number of prevented CRC cases and (v) estimate the annual rate of prevented CRC cases per 100,000 persons in the target population, that is, invited to the screening programme.

### Data

Invasive CRC (International Classification of Diseases-10th Revision code C18-C21) incidence data for the years 1997–2016 were obtained from the seven accredited general cancer registries that cover the 11 health care districts of the Emilia-Romagna Region. Two districts were covered by cancer registration only from 2005 to 2016. For the years 1997–2004, their populations were both excluded from the denominators of incidence rates. The resident population data were obtained from the Regional Administration, which collects annually the original information from all municipalities.

### Statistical methods

All observed and expected annual CRC incidence rates were age-standardised by 5-year age groups using the European standard population.

For the estimate of the expected incidence in the absence of screening, we used standard methods [32, 33]. For both sexes, we used an age-period-cohort (APC) modelling approach [34, 35] to explore the trend in CRC incidence in the years 1997–2016 by age group, time period and birth cohort, and to disentangle the effect of each of these factors. The analysis was carried out on a Lexis diagram based on 2-year time periods and 2-year age groups. We calculated the expected incidence rates using the data for all time periods before and after the introduction of the screening programme. We assumed that the screening programme may produce only a short-term non-linear period effect. Specifically, we calculated the expected incidence rates from the APC model output under the hypothesis of no screening effect, i.e. setting the values of parameters of the non-linear period effect to zero. The expected incidence represents the counterfactual scenario to be compared with the observed incidence during the years of operation of the screening programme.

Observed annual CRC incidence rates were compared with those expected with the calculation of their ratio (incidence rate ratio, IRR) with bootstrap-estimated 95% confidence interval (CI).

In order to obtain an absolute measure of the impact of the screening programme on CRC incidence [36], we calculated the annual age-standardised rate of prevented CRC cases, defined as the difference between the expected number and the observed number per 100,000 persons in the target population, with bootstrap-estimated 95% CI. This measure is the annual rate of CRC cases that are no longer observed in the target population thanks to the detection and removal of colorectal adenomas at the level of participation observed.

A sensitivity analysis was done in order to understand how the partial change in the population basis of the study occurring in 2005, with the inclusion of two health care districts previously uncovered by cancer registration (see the Data section), might affect the results. The APC modelling as well as the estimate of the expected incidence, of the IRRs, and of the annual and cumulative numbers of prevented CRC cases were replicated after the complete exclusion of the two areas from the study.

Data analysis was performed using STATA version 15.1 (Stata Corporation, College Station, TX).

## Results

On 1 January 2005, the target population included 501,826 men and 535,706 women, for a total of 1,037,532. Supplementary Table S2 shows the annual target population and the annual number of registered CRC cases over the 20 years of the study, by sex. The total number of CRC cases was 21,130 (men, *n* = 12,389; women, *n* = 8741). The proportion of death-certificate-only CRC cases was 0.1%.

The annual age-standardised rates that would be expected in the absence of screening were estimated using the APC modelling analysis. As shown in Table 1, the best-fitting model was an APC model for men and an age-period model for women. A highly significant test for interaction between sex and cohort effect (*P* < 0.001) indicated that the cohort effect differed between men and women. The models enabled identifying net changes in CRC incidence that occurred in temporal correlation with the introduction of the screening programme, assuming that a non-linear change in the period effect could be attributed to this. Further details of the modelling analysis are shown in Supplementary Table S3.

**Table 1:** Age-period-cohort modelling analysis of colorectal cancer incidence rates, by sex.

Sex and submodel^a^	Goodness of fit			Model comparison			
	Residual df	Residual deviance	Comparison	Interpretation	Change in df	Change in deviance	*P-*value^b^
Men							
1. Age	90	663.69					
2. Age-drift	89	460.86	2 versus 1	Trend (drift)	1	202.82	<0.001
3. Age-cohort	72	383.25	3 versus 2	Non-linear cohort effect	17	77.62	<0.001
4. Age-period	81	115.47	4 versus 2	Non-linear period effect	8	345.40	<0.001
5. Age-period-cohort	64	86.56	5 versus 3	Period effect adjusted for cohort	8	296.69	<0.001
			5 versus 4	Cohort effect adjusted for period	17	28.91	0.035
Women							
1. Age	90	317.11					
2. Age-drift	89	261.27	2 versus 1	Trend (drift)	1	55.83	<0.001
3. Age-cohort	72	217.84	3 versus 2	Non-linear cohort effect	17	43.43	<0.001
4. Age-period	81	91.27	4 versus 2	Non-linear period effect	8	170.00	<0.001
5. Age-period-cohort	64	73.71	5 versus 3	Period effect adjusted for cohort	8	144.13	<0.001
			5 versus 4	Cohort effect adjusted for period	17	17.56	0.417

Emilia-Romagna Region, Italy, 1997–2016.

*Df* degrees of freedom.

^a^For both sexes, five submodels (age, age-drift, age-cohort, age-period and the full age-period-cohort model) were derived. The model goodness-of-fit was evaluated based on residual deviance statistics. The age, period and birth cohort effects were derived from pairwise comparisons of the appropriate submodels. The significance of the pairwise comparisons was examined by comparing the difference in residual deviance and in degrees of freedom using the likelihood ratio test. The models 3 and 4 could not be directly compared in this way because it was not possible to construct a formal test of whether the age-cohort model was significantly better than the age-period model.

^b^Likelihood ratio test.

**Fig. 1:**
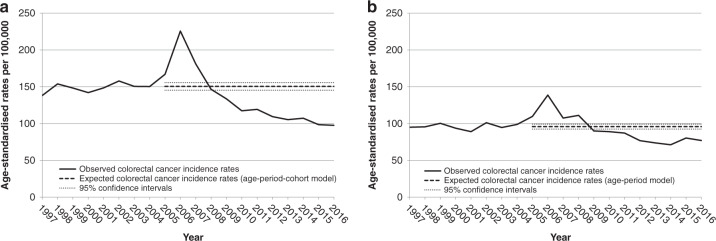
Curves of observed and expected annual colorectal cancer incidence rates. The graphs show the curve of observed annual colorectal cancer incidence rates per 100,000 persons aged 50–69 years in 1997–2016 (bold line) and the curve of rates that would be expected in 2005–2016 in the absence of the organised faecal immunochemical test screening programme (dashed line) by sex (**a** men; **b** women). The dotted lines represent the 95% confidence bands around the expected annual rates. The expected annual rates were estimated by analysing the observed annual rates in 1997–2016 with an age-period-cohort model (men) and an age-period model (women). 2005 was the year of introduction of the screening programme. 2006 was the first full year of screening. All rates were age-standardised using the European standard population. Emilia-Romagna Region, Italy, 1997–2016.

Also shown in Supplementary Table S2 are the observed annual age-standardised CRC incidence rates from 2005 to 2016 as well as those expected in the absence of screening. Both series of rates are plotted, for each sex, in Fig. 1. With respect to time trends in observed incidence, the curves were almost parallel between men (Fig. 1a) and women (Fig. 1b). In descriptive terms, the rates were fairly stable between 1997 and 2004. After an appreciable increase in 2005, a peak was observed in 2006 (the first full year of screening), followed by a deep decrease until 2013 (the eighth full year). It must be noted that in the years 2014–2016, when only minor changes occurred, the rates observed among men were nearly the same as the rates experienced by women before the screening programme was introduced. With respect to expected incidence rates between 2005 and 2016, they confirmed the stable trend observed before 2005, with an estimated average rate of 150.5 [95% CI: 145.4, 155.7] for men and 95.9 [95% CI: 92.5, 99.2] for women.

Table 2 shows the formal comparison between the observed rates and the expected ones. The observed incidence dropped significantly below the expected level in 2009 (the fifth year of screening, fourth full year) both for men and women, with an IRR of 0.88 and 0.93, respectively. The decreasing trend continued until 2013 (the eighth full year). In the subsequent years, no further significant changes occurred. Pooling the years 2013 through 2016, the IRR was 0.68 [95% CI: 0.65, 0.71] among men and 0.79 [95% CI: 0.76, 0.82] among women, for an overall figure of 0.72 [95% CI: 0.66, 0.81], equivalent to a decrease of 28%.

**Table 2:** Ratio between the observed annual colorectal cancer incidence rates per 100,000 persons aged 50–69 years in 2005–2016 and the rates that would be expected in the absence of the organised FIT screening programme, and annual and cumulative number of prevented colorectal cancer cases, by sex.

Year^a^	Men			Women		
	Incidence rate ratio [95% CI]	Annual number prevented	Cumulative number prevented	Incidence rate ratio [95% CI]	Annual number prevented	Cumulative number prevented
2005	1.11 [1.06, 1.16]	−91	−91	1.18 [1.13, 1.22]	−97	−97
2006	1.52 [1.46, 1.59]	−427	−518	1.45 [1.40, 1.51]	−249	−346
2007	1.20 [1.15, 1.25]	−163	−681	1.11 [1.07, 1.15]	−62	−408
2008	0.97 [0.93, 1.01]	21	−660	1.16 [1.12, 1.20]	−70	−478
2009	0.88 [0.85, 0.92]	77	−583	0.93 [0.89, 0.96]	32	−446
2010	0.78 [0.75, 0.81]	146	−437	0.94 [0.90, 0.97]	28	−418
2011	0.80 [0.76, 0.83]	137	−300	0.91 [0.88, 0.94]	41	−377
2012	0.73 [0.70, 0.76]	185	−115	0.80 [0.77, 0.83]	90	−287
2013	0.70 [0.67, 0.73]	204	89	0.77 [0.74, 0.80]	108	−179
2014	0.71 [0.68, 0.74]	179	268	0.74 [0.71, 0.77]	108	−71
2015	0.65 [0.62, 0.68]	220	488	0.84 [0.81, 0.87]	69	−2
2016	0.65 [0.62, 0.67]	229	717	0.81 [0.78, 0.84]	85	83

Emilia-Romagna Region, Italy, 2005–2016.

*FIT* faecal immunochemical test, *CI* (bootstrap-estimated) confidence interval.

^a^2005 was the year of introduction of the screening programme. 2006 was the first full year of screening. The annual incidence rates that would be expected in 2005–2016 in the absence of screening were estimated by analysing the observed annual rates in 1997–2016 with an age-period-cohort model for men and an age-period model for women, i.e. the models providing the best fit to the observed rates. In both models, the values of parameters of the non-linear period effect were set to zero. All rates were age-standardised using the European standard population.

In Table 2, the estimated annual number and the cumulative number of prevented CRC cases are also shown. The initial excess incidence caused the annual number to be negative until 2007 for men and 2008 for women. The cumulative number became positive in 2013 (the eighth full year) for men and in 2016 (the 11th full year) for women. From 2013 to 2016, when the incidence stabilised, the average annual number of CRC cases prevented by screening was 208 among men and 92 among women. In the first 12 years of operation, cumulatively and pooling men and women, the screening programme prevented exactly 800 CRC cases.

Table 3 shows the annual age-standardised rate of prevented CRC cases per 100,000 persons in the target population. In 2016, the rate was 52.8 per 100,000 men and 18.9 per 100,000 women.

**Table 3:** Annual screening-attributable rate of prevented colorectal cancer cases defined as the difference between the expected number and the observed number per 100,000 persons aged 50–69 years in the target population of the organised FIT screening programme, by sex.

Year^a^	Annual screening-attributable rate of prevented colorectal cancer cases [95% CI]	
	Men	Women
2005	−16.4 [−22.4, −9.9]	−13.9 [−17.4, −10.3]
2006	−76.1 [−82.1, −69.6]	−42.9 [−46.4, −39.3]
2007	−31.3 [−37.3, −24.8]	−11.4 [−14.9, −7.8]
2008	3.1 [−2.8, 9.7]	−15.3 [−18.8, −11.7]
2009	16.9 [10.9, 23.4]	6.0 [2.5, 9.6]
2010	32.6 [26.6, 39.1]	5.9 [2.4, 9.5]
2011	31.5 [25.5, 38.0]	8.5 [5.0, 12.1]
2012	40.5 [34.5, 47.0]	18.8 [15.3, 22.4]
2013	45.4 [39.5, 51.9]	22.0 [18.5, 25.6]
2014	42.9 [36.9, 49.4]	24.5 [21.0, 28.1]
2015	51.7 [45.8, 58.3]	15.0 [11.5, 18.6]
2016	52.8 [46.8, 59.3]	18.9 [15.4, 22.5]

Emilia-Romagna Region, Italy, 2005–2016.

*FIT* faecal immunochemical test, *CI* (bootstrap-estimated) confidence interval.

^a^2005 was the year of introduction of the screening programme. 2006 was the first full year of screening. The expected number is the number of incident colorectal cancer cases that would be expected between 2005 and 2016 in the absence of screening, estimated by analysing the observed annual rates in 1997–2016 with an age-period-cohort model for men and an age-period model for women, i.e. the models providing the best fit to the observed rates. In both models, the values of parameters of the non-linear period effect were set to zero. All rates were age-standardised using the European standard population.

For sensitivity analysis purposes, all of the above estimates were replicated after exclusion of the two health care districts uncovered by cancer registration until 2004, which accounted for a total population of 282,051 in 2005 and a total 2297 CRC cases. The results of the APC modelling are shown in Supplementary Table S4. An APC model for men and an age-period model for women were confirmed to be the best-fitting models. The comparison between the observed rates and the expected ones and the estimated annual and cumulative numbers of prevented CRC cases are shown in Supplementary Table S5. Virtually no changes versus the original analysis were found. In the pooled years 2013–2016, the IRR was 0.68 [95% CI: 0.65, 0.71] for men, 0.78 [95% CI: 0.76, 0.81] for women and 0.72 [95% CI: 0.65, 0.81] for both sexes combined. In the first 12 years, the number of CRC cases prevented by the screening programme increased moderately to 901 (both sexes combined).

## Discussion

This study explored the effects of a FIT screening programme on annual CRC incidence rates in its dynamic target population. The main findings were that: (i) the decrease in annual rates became significant during the 4th full year of operation; (ii) it continued for both sexes until the eighth year and then roughly stabilised; (iii) in the last four study years the overall IRR for both sexes combined was 0.72; (iv) in each of the same four years the programme prevented an average annual number of 208 CRC cases among men and 92 among women, which were equivalent to about 53 cases every 100,000 men invited to screening and 19 cases every 100,000 women invited to screening and (vi) in the first 12 years of the programme the cumulative number of prevented CRC cases was 800. Another interesting outcome was that the rates observed among men in the years 2014–2016, when only minor incidence changes occurred, were nearly the same as those seen among women before the introduction of the programme.

With respect to the latency time of the preventive effect, we started to observe significant incidence changes in 2009, that is, the 4th full year of screening. In once-only sigmoidoscopy trials, the latency time of the effect on distal CRC was longer, as a decrease in cumulative incidence was discernible only after 5–6 years since randomisation [6, 8, 9]. This difference depends mainly on the fact that the cumulative rate in a cohort study includes the initial prevalence peak. The cumulative incidence provides a measure of the overall risk of disease. Annual rates, conversely, are more informative of the public health impact of screening (in particular, of the annual CRC surgery workload) and of research issues (in particular, the lead time on prevented CRC cases).

As specifically regards the lead time, the second key finding of this study was that the incidence decrease continued until the eighth full year of screening. As suggested by studies of cervical cancer screening [32, 33], the rates were expected to decrease for a time span that is an approximate measure of the lead time of CRC cases prevented by the detection and treatment of precancerous lesions. The temporal duration of the adenoma-carcinoma sequence and, thus, the potential lead time of prevented CRC cases are 10–15 years in most instances [37]. Given the low sensitivity of FIT for initial adenomas [38], however, lead times of this length are unlikely to be generated to a significant extent. This explains why incidence rates in this study stabilised within less than 10 years of screening.

The third, and most important, finding of this study was that the screening programme was associated with an overall 28% decrease in annual CRC incidence. The rate decreased more rapidly for men and the magnitude of the impact in the last study years was greater, that is, 32% versus 21%. For women, a longer latency time is compatible with a lower growth rate of the disease. Preclinical studies have suggested a protective role for estrogens both in the initiation and in the progression of CRC [39], although their role remains controversial. With respect to the final impact on incidence, the observed between-sex difference is in accordance with the results of a previous study—from the same screening programme—on the proportional incidence of interval CRC, i.e. the age-standardised ratio between the observed incidence in a cohort of men and women with negative FIT result and the incidence that would be expected in the absence of screening (estimated with APC models) [31]. We found figures of 0.06 among men and 0.17 among women in the first interval year and, respectively, 0.21 and 0.28 in the second year, indicating that repeated FIT screening is less sensitive for adenoma and early invasive CRC in the female population. In absolute terms, however, the level of sensitivity of FIT is considered high in both sexes [40].

To further characterise the impact of the screening programme on CRC incidence, we calculated the annual rate of prevented CRC cases per 100,000 persons in the target population (that is, the invited population) [36]. This measure indicates the annual rate of CRC cases that are no longer diagnosed nor treated as a result of the detection and removal of colorectal adenomas. If compared, in particular, with the annual incidence rates of some common malignancies, it may provide a straightforward quantification of the preventive effect of the programme at the public health level. For example, the figure observed in the male target population in 2016, i.e. 52.8 per 100,000, was nearly equal to the whole annual age-standardised (European standard population) incidence rate of cutaneous malignant melanoma in the same population and in the same year, i.e. 50.1 per 100,000 (this rate was calculated using data from the local Romagna Cancer Registry).

The findings of the present intention-to-screen study are in keeping with a recently published cohort study from the same screening programme [26]. Comparing attenders with non-attenders, the CRC incidence at 11 years of follow-up was 33% lower among men and 21% lower among women. Both estimates were self-selection-adjusted. The consistency of their results, which are based on different designs, provides confidence in the robustness of the conclusions of both studies. Comparisons with other previous observational studies, conversely, should be made with caution. In a registry-based study with a design similar to ours but with a shorter time period of observation and much lower statistical power, no apparent effect was found [41]. In two studies of limited size, the decrease in cumulative CRC rate was 22% among screening participants relative to non-participants [23] and 10% in the invited population relative to a historical control population [24]. Again, the latter modest result can be explained by a short duration of follow-up.

In their simulation model study, Lew et al. estimated the effects of the biennial Australian National Bowel Cancer Screening Programme (NBCSP) on a population of people invited between 50 and 74 years of age according to different assumptions as to the participation rate [2]. The programme was fully rolled-out in as many as 15 years (2006–2020). The simulation model covered the years 2015–2040. Based on our findings as well as literature data on other screening models [32, 33, 42, 43], the effects of the NBCSP on CRC incidence during 2015–2040 are expected to reach the steady-state rapidly and then stabilise. By implication, the incidence reduction estimated by Lew et al. is fairly comparable with our estimate of the decrease in annual CRC incidence rates after eight full years of operation. With a participation rate of 40% and 60%, according to the Australian study, the NBCSP is expected to reduce CRC incidence by 23% and, respectively, 33% [2]. Our figures, i.e. a participation rate of ~50% and an incidence reduction of 28%, are intermediate between these estimates and—consequently—quite consistent with them.

Our results also corroborate those of a previous Italian national incidence study covering 48 local population subsets for a total of 36 millions [44]. In the age range 50–69 years, CRC rates over the last two decades showed a significant increase in both sexes until 2006–2007, a significant decrease until 2010, and a stabilisation thereafter. This 3-phase incidence pattern—similar to the one seen in our data—was interpreted by the authors to mirror the effect of the introduction of several local FIT screening programmes. It must be noted, however, that the previous study covered a little more than a decade of observation and simply described time trends in incidence. It did not establish a formal temporal correlation between these and the introduction of local screening programmes, which were started in different years and had different paces of implementation and varying participation rates, nor did it attempt to measure the decrease in CRC incidence observed in 2010 and after. Consequently, our results add substantial information to previous ones.

Some methodological issues of this study deserve mentioning. First, a temporal correlation does not formally prove a causal link. In our data, however, there are multiple consistent circumstantial evidences for a cause-effect relationship between the introduction of screening and the observed decrease in CRC incidence, namely: (i) before the introduction of the programme, CRC rates were stable, which provided the ideal conditions for the relationship to be assessed; (ii) the time lag between the two events was very short thanks to the fact that the target population was rapidly saturated by invitations—a key issue of temporal correlation studies between screening and incidence changes [45]; (iii) the incidence changes followed the same temporal pattern in both sexes but were more pronounced in the male population, in accordance with the local study on the sensitivity of FIT by sex [31]; (iv) the atypical shape of incidence curves, with a pronounced and transient peak immediately followed by a deep drop, was not compatible with changes in exposure to risk factors for CRC and (v) the APC modelling analysis showed incidence changes occurring after 2005 that might be related to an intervening external factor [35]. For these facts, we do not see a comprehensive explanation other than the introduction of the screening programme in 2005.

Second, incidence estimates are prone to biases. The APC modelling, however, is considered the reference method for estimating the incidence rates that would be expected in the absence of a screening activity, that is, the true incidence rates underlying the observed rates that are distorted [32, 33]. A mention should be made of the fact that the best-fitting models were an APC model for men and an age-period model for women. The absence of a significant cohort effect among women might well be due to the smaller numbers. However, partial differences in the aetiology of CRC and in the level of exposure to risk factors cannot be excluded. It appears that this finding merits further consideration.

Third, the intention-to-screen approach allows to estimate the impact of a screening programme on the whole invited population, whatever the extent to which it was actually and successfully screened, but underestimates the magnitude of the effect that occurs among the participants. On the other hand, applying the intention-to-screen principle yields an unbiased and more accurate estimate of the effectiveness of the intervention under real-world conditions.

Fourth, two health care districts of the study area were covered by cancer registration only from 2005 to 2016. For the years 1997–2004, their populations were excluded from the calculation of incidence rates. A sensitivity analysis was done in order to determine the extent to which the partial modification of the population basis of the study in 2005 might affect the results. Virtually no changes in the IRRs were observed after the complete exclusion of the two areas from the analysis.

As a related problem, the completeness of CRC registration in the study area is not absolute. It must be considered, however, that the death-certificate-only index decreases gradually over the years [46], and that the registries participating in this study had been operating for a period ranging from 6 to 19 years before 1997. This explains why the proportion of death-certificate-only CRC cases in the study dataset was as low as 0.1%, which is equivalent to saying that the related potential biases in incidence trends were less than marginal.

The last methodological issue to be highlighted is that some caution is required in extrapolating our results to a screening population with different characteristics, especially with respect to age distribution and prevalence of disease, and to a different faecal haemoglobin concentration cut-off value. A German study, for example, showed that lowering the cut-off from ≥20 μg Hb/g faeces to ≥9 μg Hb/g faeces may increase the sensitivity for advanced colorectal neoplasms from ~34 to 49%, although this would be achieved at the expense of a doubling of FIT positivity rate, from 8 to 16% and a substantial loss in specificity, from 96 to 89% [47].

In conclusion, this study provided multiple circumstantial but consistent proofs of a causal relationship between the introduction of a public health FIT screening programme and a stable 28% overall decrease in annual CRC incidence rates after eight full years of operation. The demonstration of this effect on incidence reinforces the rationale of ongoing and future programmes.

## Supplementary information

Additional information

Authorship agreement

aj-checklist

Consortium authorship status

## Data Availability

The data used in this study are available from the corresponding author upon reasonable request.

## References

[1] Brenner H, Hoffmeister M, Stegmaier C, Brenner G, Altenhofen L, Haug U (2007). Risk of progression of advanced adenomas to colorectal cancer by age and sex: estimates based on 840,149 screening colonoscopies. Gut..

[2] Lew JB, St John DJB, Xu XM, Greuter MJE, Caruana M, Cenin DR (2017). Long-term evaluation of benefits, harms, and cost-effectiveness of the National Bowel Cancer Screening Program in Australia: a modelling study. Lancet Public Health.

[3] Lew JB, Feletto E, Wade S, Caruana M, Kang YJ, Nickson C (2019). Benefits, harms and cost-effectiveness of cancer screening in Australia: an overview of modelling estimates. Public Health Res Pract.

[4] Jørgensen OD, Kronborg O, Fenger C, Rasmussen M (2007). Influence of long-term colonoscopic surveillance on incidence of colorectal cancer and death from the disease in patients with precursors (adenomas). Acta Oncol.

[5] Kahi CJ, Imperiale TF, Juliar BE, Rex DK (2009). Effect of screening colonoscopy on colorectal cancer incidence and mortality. Clin Gastroenterol Hepatol.

[6] Atkin WS, Edwards R, Kralj-Hans I, Wooldrage K, Hart AR, Northover JM (2010). Once-only flexible sigmoidoscopy screening in prevention of colorectal cancer: a multicentre randomised controlled trial. Lancet..

[7] Brenner H, Chang-Claude J, Seiler CM, Rickert A, Hoffmeister M (2011). Protection from colorectal cancer after colonoscopy: a population-based, case-control study. Ann Intern Med.

[8] Segnan N, Armaroli P, Bonelli L, Risio M, Sciallero S, Zappa M (2011). Once-only sigmoidoscopy in colorectal cancer screening: follow-up findings of the Italian randomized controlled trial – SCORE. J Natl Cancer Inst.

[9] Schoen RE, Pinsky PF, Weissfeld JL, Yokochi LA, Church T, Laiyemo AO (2012). Colorectal-cancer incidence and mortality with screening flexible sigmoidoscopy. N Engl J Med.

[10] Mandel JS, Church TR, Bond JH, Ederer F, Geisser MS, Mongin SJ (2000). The effect of fecal occult-blood screening on the incidence of colorectal cancer. N Engl J Med.

[11] Kewenter J, Brevinge H, Engarås B, Haglind E, Ahrén C (1994). Results of screening, rescreening, and follow-up in a prospective randomized study for detection of colorectal cancer by fecal occult blood testing: results for 68,308 subjects. Scand J Gastroenterol.

[12] Hardcastle JD, Chamberlain JO, Robinson MH, Moss SM, Amar SS, Balfour TW (1996). Randomised controlled trial of faecal-occult-blood screening for colorectal cancer. Lancet..

[13] Kronborg O, Fenger C, Olsen J, Jørgensen OD, Søndergaard O (1996). Randomised study of screening for colorectal cancer with faecal-occult-blood test. Lancet..

[14] Faivre J, Dancourt V, Lejeune C, Tazi MA, Lamour J, Gerard D (2004). Reduction in colorectal cancer mortality by fecal occult blood screening in a French controlled study. Gastroenterology..

[15] Lansdorp-Vogelaar I, von Karsa L Introduction. In: Segnan N, Patnick J, von Karsa L (eds). European guidelines for quality assurance in colorectal cancer screening and diagnosis, 1st edn. Luxembourg: Publications Office of the European Union; 2010, pp 1–31.

[16] Robertson DJ, Lee JK, Boland CR, Dominitz JA, Giardiello FM, Johnson DA (2017). Recommendations on fecal immunochemical testing to screen for colorectal neoplasia: a consensus statement by the US Multi-Society Task Force on Colorectal Cancer. Gastroenterology..

[17] Rex DK, Boland CR, Dominitz JA, Giardiello FM, Johnson DA, Kaltenbach T (2017). Colorectal cancer screening: recommendations for physicians and patients from the U.S. Multi-Society Task Force on Colorectal Cancer. Am J Gastroenterol.

[18] van Rossum LG, van Rijn AF, Laheij RJ, van Oijen MG, Fockens P, van Krieken HH (2008). Random comparison of guaiac and immunochemical fecal occult blood tests for colorectal cancer in a screening population. Gastroenterology..

[19] Lee JK, Liles EG, Bent S, Levin TR, Corley DA (2014). Accuracy of fecal immunochemical tests for colorectal cancer: systematic review and meta-analysis. Ann Intern Med.

[20] Lin JS, Piper MA, Perdue LA, Rutter CM, Webber EM, O’Connor E (2016). Screening for colorectal cancer: updated evidence report and systematic review for the US Preventive Services Task Force. JAMA..

[21] Zauber AG (2015). The impact of screening on colorectal cancer mortality and incidence: has it really made a difference?. Dig Dis Sci.

[22] Greuter MJ, Demirel E, Lew JB, Berkhof J, Xu XM, Canfell K (2016). Long-term impact of the Dutch colorectal cancer screening program on cancer incidence and mortality: model-based exploration of the serrated pathway. Cancer Epidemiol Biomark Prev.

[23] Ventura L, Mantellini P, Grazzini G, Castiglione G, Buzzoni C, Rubeca T (2014). The impact of immunochemical faecal occult blood testing on colorectal cancer incidence. Dig Liver Dis.

[24] Giorgi Rossi P, Vicentini M, Sacchettini C, Di Felice E, Caroli S, Ferrari F (2015). Impact of screening program on incidence of colorectal cancer: a cohort study in Italy. Am J Gastroenterol.

[25] Baldacchini F, Bucchi L, Giuliani O, Mancini S, Ravaioli A, Vattiato R, et al. Effects of attendance to an organized fecal immunochemical test screening program on the risk of colorectal cancer: an observational cohort study. Clin Gastroenterol Hepatol. 2022. 10.1016/j.cgh.2022.01.053. 10.1016/j.cgh.2022.01.053 35144023

[26] Levin TR, Corley DA, Jensen CD, Schottinger JE, Quinn VP, Zauber AG (2018). Effects of organized colorectal cancer screening on cancer incidence and mortality in a large community-based population. Gastroenterology..

[27] Mancini S, Ravaioli A, Falcini F, Giuliani O, Corradini R, De Girolamo G, et al. Strategies for delivery of faecal occult blood test kits and participation to colorectal cancer screening in the Emilia-Romagna Region of Italy. Eur J Cancer Care (Engl). 2018;27:e12631. 10.1111/ecc.12631 28032381

[28] Sassoli de Bianchi P, Campari C, Mancini S, Giuliani O, Landi P, Paterlini L (2013). Colonoscopic surveillance of first-degree relatives of colorectal cancer patients in a faecal occult blood screening programme. Cancer Epidemiol.

[29] Zorzi M, de’ Bianchi PS, Grazzini G, Senore C (2007). Quality indicators for the evaluation of colorectal cancer screening programmes. Epidemiol Prev.

[30] Baldacchini F, Bucchi L, Giuliani O, Mancini S, Ravaioli A, Vattiato R (2021). Results of compliant participation in five rounds of fecal immunochemical test screening for colorectal cancer. Clin Gastroenterol Hepatol.

[31] Mancini S, Bucchi L, Giuliani O, Ravaioli A, Vattiato R, Baldacchini F (2020). Proportional incidence of interval colorectal cancer in a large population-based faecal immunochemical test screening programme. Dig Liver Dis.

[32] Vaccarella S, Franceschi S, Engholm G, Lönnberg S, Khan S, Bray F (2014). 50 years of screening in the Nordic countries: quantifying the effects on cervical cancer incidence. Br J Cancer.

[33] Bucchi L, Baldacchini F, Mancini S, Ravaioli A, Giuliani O, Vattiato R (2019). Estimating the impact of an organised screening programme on cervical cancer incidence: a 26-year study from northern Italy. Int J Cancer.

[34] Clayton D, Schifflers E (1987). Models for temporal variation in cancer rates. I: age-period and age-cohort models. Stat Med.

[35] Clayton D, Schifflers E. Models for temporal variation in cancer rates. II: age-period-cohort models. Stat Med. 1987;6:469–81. 10.1002/sim.4780060406 3629048

[36] Wacholder S (2005). The impact of a prevention effort on the community. Epidemiology..

[37] Day DW, Morson BC (1978). The adenoma-carcinoma sequence. Major Probl Pathol.

[38] Lansdorp-Vogelaar I, Goede SL, Bosch LJW, Melotte V, Carvalho B, van Engeland M (2018). Cost-effectiveness of high-performance biomarker tests vs fecal immunochemical test for noninvasive colorectal cancer screening. Clin Gastroenterol Hepatol.

[39] Barzi A, Lenz AM, Labonte MJ, Lenz HJ (2013). Molecular pathways: estrogen pathway in colorectal cancer. Clin Cancer Res.

[40] Zorzi M, Fedato C, Grazzini G, Stocco FC, Banovich F, Bortoli A (2011). High sensitivity of five colorectal screening programmes with faecal immunochemical test in the Veneto Region, Italy. Gut..

[41] Zorzi M, Fedeli U, Schievano E, Bovo E, Guzzinati S, Baracco S (2015). Impact on colorectal cancer mortality of screening programmes based on the faecal immunochemical test. Gut..

[42] Swedish Organised Service Screening Evaluation Group. (2007). Effect of mammographic service screening on stage at presentation of breast cancers in Sweden. Cancer..

[43] Tabár L, Yen AM, Wu WY, Chen SL, Chiu SY, Fann JC (2015). Insights from the breast cancer screening trials: how screening affects the natural history of breast cancer and implications for evaluating service screening programs. Breast J.

[44] Zorzi M, Dal Maso L, Francisci S, Buzzoni C, Rugge M, Guzzinati S (2019). Trends of colorectal cancer incidence and mortality rates from 2003 to 2014 in Italy. Tumori.

[45] Moss SM, Nyström L, Jonsson H, Paci E, Lynge E, Njor S (2012). The impact of mammographic screening on breast cancer mortality in Europe: a review of trend studies. J Med Screen.

[46] Brenner H, Jansen L (2013). Determinants and interpretation of death certificate only proportions in the initial years of newly established cancer registries. Eur J Cancer.

[47] Brenner H, Werner S (2017). Selecting a cut-off for colorectal cancer screening with a fecal immunochemical test. Clin Transl Gastroenterol.

