# Supplementary material for: How a faecal immunochemical test screening programme changes annual colorectal cancer incidence rates: an Italian intention-to-screen study
Source: Br J Cancer. 2022 Apr 20;127(3):541–8. doi: 10.1038/s41416-022-01813-7 (PMC9345854; doi:10.1038/s41416-022-01813-7)
Supplement: Supplementary file 1 — Additional information [file 41416_2022_1813_MOESM1_ESM.pdf]

British Journal of Cancer

How a faecal immunochemical test screening programme changes annual colorectal cancer incidence rates: an Italian intention-to-screen study

Lauro Bucchi[1], Silvia Mancini[1✉], Flavia Baldacchini[1], Alessandra Ravaioli[1], Orietta Giuliani[1], Rosa Vattiato[1], Paolo Giorgi Rossi[2], Cinzia Campari[3], Debora Canuti[4], Enza Di Felice[5], Priscilla Sassoli de Bianchi[5], Stefano Ferretti[6], Nicoletta Bertozzi[5], Annibale Biggeri[7] and Fabio Falcini[1,8], on behalf of the Emilia-Romagna Region Workgroup for Colorectal Screening Evaluation

[1]Romagna Cancer Registry, Romagna Cancer Institute, *Istituto Romagnolo per lo Studio dei Tumori "Dino Amadori", IRST, IRCCS*, Meldola, Forlì, Italy. [2]Epidemiology Unit, Azienda Unità Sanitaria Locale – IRCCS di Reggio Emilia, Reggio Emilia, Italy. [3]Cancer Screening Unit, Azienda Unità Sanitaria Locale – IRCCS di Reggio Emilia, Reggio Emilia, Italy. [4]Cancer Screening Unit, Local Health Authority, Rimini, Italy. [5]Department of Health, Regional Administration, Emilia-Romagna Region, Bologna, Italy. [6]University of Ferrara and Local Health Authority, Ferrara, Italy. [7]Unit of Biostatistics, Epidemiology and Public Health, Department of Cardiac, Thoracic, Vascular Sciences and Public Health, University of Padua, Padua, Italy. [8]Cancer Prevention Unit, Local Health Authority, Forlì, Italy. ✉email: silvia.mancini@irst.emr.it

ADDITIONAL INFORMATION

Supplementary Table S1
Supplementary Table S2
Supplementary Table S3
Supplementary Table S4
Supplementary Table S5

Supplementary Table S1. Target population aged 50-69 years and main average annual performance measures of the organised FIT screening programme in three quadrennial time periods, by sex. Emilia-Romagna Region, Italy, 2005-2016.

	**Measure**	**Men**			**Women**		
		**2005-2008**	**2009-2012**	**2013-2016**	**2005-2008**	**2009-2012**	**2013-2016**
Population							
	Annual target population, *n*	253,424	262,296	278,507	269,698	280,101	298,248
	Invited, *n*	220,599	252,235	268,963	237,285	271,546	289,041
	Attenders, *n* (% invited)	107,577 (48.8)	126,139 (50.0)	133,101 (49.5)	124,769 (52.6)	145,823 (53.7)	153,888 (53.2)
	Recalled, *n* (% attenders)	7155 (6.7)	6108 (4.8)	6904 (5.2)	5551 (4.4)	5177 (3.6)	6258 (4.1)
First FIT							
	Recalled, *n* (% attenders)	5545 (7.1)	1796 (5.9)	1557 (5.8)	4181 (4.7)	1338 (4.0)	1267 (4.4)
	Undergoing colonoscopy,[a] *n* (% recalled)	4554 (82.1)	1459 (81.2)	1252 (80.4)	3322 (79.5)	1068 (79.8)	1001 (79.0)
	With non-advanced adenoma, *n* (per 1000 attenders)	702 (9.0)	206 (6.7)	186 (7.0)	400 (4.5)	122 (3.7)	116 (4.0)
	With advanced adenoma,[b] *n* (per 1000 attenders)	1867 (24.0)	545 (17.8)	402 (15.0)	1021 (11.5)	276 (8.3)	209 (7.2)
	With CRC, *n* (per 1000 attenders)	348 (4.5)	73 (2.4)	46 (1.7)	214 (2.4)	53 (1.6)	40 (1.4)
Subsequent FITs							
	Recalled, *n* (% attenders)	3221 (5.4)	4312 (4.5)	5346 (5.0)	2741 (3.8)	3839 (3.4)	4991 (4.0)
	Undergoing colonoscopy,[a] *n* (% recalled)	2662 (82.6)	3615 (83.8)	4401 (83.3)	2185 (79.7)	3144 (81.9)	4010 (80.3)
	With non-advanced adenoma, *n* (per 1000 attenders)	521 (8.7)	667 (7.0)	885 (8.3)	318 (4.4)	431 (3.8)	572 (4.6)
	With advanced adenoma,[b] *n* (per 1000 attenders)	807 (13.6)	1093 (11.4)	1098 (10.3)	452 (6.3)	670 (6.0)	704 (5.6)
	With CRC, *n* (per 1000 attenders)	111 (1.9)	128 (1.3)	120 (1.1)	82 (1.1)	104 (0.9)	102 (0.8)

FIT faecal immunochemical test, *CRC* colorectal cancer.

[a]Patients undergoing computed tomographic colonography after incomplete colonoscopy were excluded.

[b]Defined as an adenoma >10 mm in diameter or with villous histology or high-grade dysplasia.

Supplementary Table S2. Target population aged 50-69 years, observed annual colorectal cancer incidence (numbers and rates per 100,000 persons) in 1997-2016, and annual rates that would be expected in 2005-2016 in the absence of the organised FIT screening programme, by sex. Emilia-Romagna Region, Italy, 1997-2016.

**Year**[a]	**Men**				**Women**			
	**Population, *n***	**Observed number**	**Observed rate, per 100,000**	**Expected rate [95% CI], per 100,000**	**Population, *n***	**Observed number**	**Observed rate, per 100,000**	**Expected rate [95% CI], per 100,000**
1997	340,781	511	138.1	NA	365,328	366	95.0	NA
1998	343,217	574	153.9	NA	366,452	374	95.6	NA
1999	345,897	549	148.3	NA	368,281	393	100.3	NA
2000	347,648	531	142.1	NA	369,563	365	93.7	NA
2001	348,257	554	148.5	NA	369,591	349	89.0	NA
2002	348,815	589	157.9	NA	369,556	397	101.2	NA
2003	363,078	584	150.5	NA	384,406	387	94.6	NA
2004	500,636	811	150.3	NA	534,150	561	98.8	NA
2005	501,826	909	167.0	150.5 [145.4, 155.7]	535,706	645	109.7	95.9 [92.5, 99.2]
2006	503,779	1246	225.7	150.5 [145.4, 155.7]	536,635	797	138.7	95.9 [92.5, 99.2]
2007	509,152	992	181.1	150.5 [145.4, 155.7]	540,450	615	107.5	95.9 [92.5, 99.2]
2008	410,594	644	146.6	150.5 [145.4, 155.7]	433,607	511	111.2	95.9 [92.5, 99.2]
2009	413,169	588	133.6	150.5 [145.4, 155.7]	437,382	411	89.9	95.9 [92.5, 99.2]
2010	419,320	522	117.4	150.5 [145.4, 155.7]	444,764	419	89.0	95.9 [92.5, 99.2]
2011	423,809	534	119.3	150.5 [145.4, 155.7]	450,554	409	87.1	95.9 [92.5, 99.2]
2012	430,173	493	109.4	150.5 [145.4, 155.7]	458,363	367	76.8	95.9 [92.5, 99.2]
2013	436,796	485	105.3	150.5 [145.4, 155.7]	465,978	357	73.7	95.9 [92.5, 99.2]
2014	394,252	440	107.3	150.5 [145.4, 155.7]	419,393	309	71.2	95.9 [92.5, 99.2]
2015	403,519	412	98.5	150.5 [145.4, 155.7]	428,625	357	80.4	95.9 [92.5, 99.2]
2016	413,410	421	97.6	150.5 [145.4, 155.7]	438,539	352	77.0	95.9 [92.5, 99.2]

FIT faecal immunochemical test, *CI* confidence interval, *NA* not applicable.

[a]2005 was the year of introduction of the screening programme. 2006 was the first full year of screening. The annual incidence rates that would be expected in 2005-2016 in the absence of screening were estimated by analysing the observed annual rates in 1997-2016 with an age-period-cohort model for men and an age-period model for women, i.e., the models providing the best fit to the observed rates. In both models, the values of parameters of the non-linear period effect were set to zero. All rates were age-standardised using the European standard population.

Supplementary Table S3. Parameters of the age-period-cohort models selected for estimating the colorectal cancer incidence rates that would be expected in the absence of the organised FIT screening programme, by sex. Emilia-Romagna Region, Italy, 1997-2016.

Sex	Parameters	Rate/IRR[a]	Test statistic	*P* value	95% CI
Men					
	Age at diagnosis (years)				
	50-51	62.0	-156.28	<0.001	56.5, 68.0
	52-53	70.6	-165.20	<0.001	64.8, 77.0
	54-55	85.7	-176.50	<0.001	79.2, 92.7
	56-57	118.5	-194.39	<0.001	110.7, 126.9
	58-59	135.9	-196.47	<0.001	127.2, 145.1
	60-61	171.4	-202.04	<0.001	161.1, 182.3
	62-63	191.8	-200.78	<0.001	180.4, 203.9
	64-65	238.7	-205.91	<0.001	225.4, 252.9
	66-67	276.2	-201.73	<0.001	260.8, 292.4
	68-69	318.1	-199.22	<0.001	300.6, 336.6
	Non-linear period effect				
	<2005	1.00			
	≥2005	1.49	14.57	<0.001	1.42, 1.58
	Post-screening drift	0.85	-20.58	<0.001	0.84, 0.87
	Non-linear cohort effect				
	cohort 1935 to 1939	0.97	-1.10	0.273	0.92, 1.02
	cohort 1959 to 1965	1.12	2.06	0.040	1.01, 1.25
	ln(Y)	1(exposure)			
Women					
	Age at diagnosis (years)				
	50-51	54.8	-161.47	<0.001	50.1, 60.1
	52-53	58.0	-161.98	<0.001	53.0, 63.5
	54-55	65.3	-166.64	<0.001	59.9, 71.2
	56-57	85.7	-180.44	<0.001	79.4, 92.5
	58-59	97.4	-186.26	<0.001	90.6, 104.8
	60-61	106.3	-189.33	<0.001	99.0, 114.1
	62-63	116.3	-193.84	<0.001	108.6, 124.5
	64-65	136.1	-201.41	<0.001	127.7, 145.2
	66-67	144.9	-203.22	<0.001	136.1, 154.4
	68-69	163.5	-207.64	<0.001	153.9, 173.7
	Period effect				
	<2005	1.00			
	2005-2006	1.31	8.62	<0.001	1.24, 1.40
	2007-2008	1.13	3.60	<0.001	1.06, 1.21
	2009-2010	0.93	-1.78	0.075	0.86, 1.01
	2011-2012	0.86	-3.91	<0.001	0.79, 0.93
	2013-2014	0.76	-6.58	<0.001	0.69, 0.82
	2015-2016	0.82	-4.72	<0.001	0.76, 0.89
	ln(Y)	1(exposure)			

IRR incidence rate ratio, *CI* confidence interval.

The authors fitted a series of Poisson regression models on the number of case events and person-years. The models were as follows: age (10 categorical classes: 50-51, 52-53, 54-55, 56-57, 58-59, 60-61, 62-63, 64-65, 66-67, and 68-69 years), age-drift (linear ordinal variable indexing the 10 biennal periods), age-period (10 categorical classes: 1997-1998, 1999-2000, 2001-2002, 2003-2004, 2005-2006, 2007-2008, 2009-2010, 2011-2012, 2013-2014, and 2015-2016), age-cohort (19 categorical birth cohorts from 1928-1931 to 1964-1967), and age-period-cohort. Nested models were compared with the likelihood ratio test. The authors subsequently specified a more parsimonious model according to the previous model selection strategy, which considered a modification of the drift since the introduction of the screening programme. For men, the final model included an interaction term between drift and a dummy variable (0 before, 1 after) for the years of the screening programme. The output of the final model is reported above. Note that, instead of specifying the interaction as main effects and interaction terms, the authors preferred to code a dummy variable for the baseline incidence before the introduction of the screening programme and an ordinal variable for the linear trend after the introduction [1]. The two specifications are algebraically equivalent. For women, the final model included only the linear and non-linear period effects. The linear trend before the introduction of the screening programme was essentially flat. Accordingly, the pre-screening period was used as the reference category.

[a]Incidence rates per 100,000 persons are provided for age at diagnosis. IRRs are provided for period, cohort and drift (when these variables are in the model).

REFERENCE

1. Clayton D, Hills M. Statistical models in epidemiology. Oxford, UK: Oxford University Press; 1993, pp 256-257.

Supplementary Table S4. Age-period-cohort modelling analysis of colorectal cancer incidence rates, by sex. For sensitivity analysis purposes, the two health care districts that were not covered by cancer registration until 2004 were excluded. Emilia-Romagna Region, Italy, 1997-2016.

**Sex and submodel**[a]	**Goodness of fit**			**Model comparison**			
	**Residual df**	**Residual deviance**	**Comparison**	**Interpretation**	**Change in df**	**Change in deviance**	***P* value**[b]
Men							
1. Age	90	585.10					
2. Age-drift	89	410.71	2 versus 1	Trend (drift)	1	174.39	<0.001
3. Age-cohort	72	346.22	3 versus 2	Non-linear cohort effect	17	64.49	<0.001
4. Age-period	81	104.25	4 versus 2	Non-linear period effect	8	306.46	<0.001
5. Age-period-cohort	64	74.50	5 versus 3	Period effect adjusted for cohort	8	271.72	<0.001
			5 versus 4	Cohort effect adjusted for period	17	29.75	0.028
Women							
1. Age	90	274.29					
2. Age-drift	89	229.96	2 versus 1	Trend (drift)	1	44.33	<0.001
3. Age-cohort	72	193.26	3 versus 2	Non-linear cohort effect	17	36.70	0.004
4. Age-period	81	87.06	4 versus 2	Non-linear period effect	8	142.90	<0.001
5. Age-period-cohort	64	69.68	5 versus 3	Period effect adjusted for cohort	8	123.59	<0.001
			5 versus 4	Cohort effect adjusted for period	17	17.38	0.429

Df degrees of freedom.

[a]For both sexes, five submodels (age, age-drift, age-cohort, age-period, and the full age-period-cohort model) were derived. The model goodness-of-fit was evaluated based on residual deviance statistics. The age, period, and birth cohort effects were derived from pairwise comparisons of the appropriate sub-models. The significance of the pairwise comparisons was examined by comparing the difference in residual deviance and in degrees of freedom using the likelihood ratio test. The models 3 and 4 could not be directly compared in this way because it was not possible to construct a formal test of whether the age-cohort model was significantly better than the age-period model.

[b]Likelihood ratio test.

Supplementary Table S5. Ratio between the observed annual colorectal cancer incidence rates per 100,000 persons aged 50-69 years in 2005-2016 and the rates that would be expected in the absence of the organised FIT screening programme, and annual and cumulative number of prevented colorectal cancer cases, by sex. For sensitivity analysis purposes, the two health care districts that were not covered by cancer registration until 2004 were excluded. Emilia-Romagna Region, Italy, 2005-2016.

Year[a]	Men			Women		
	**Incidence rate ratio [95% CI]**	**Annual number prevented**	**Cumulative number prevented**	**Incidence rate ratio [95% CI]**	**Annual number prevented**	**Cumulative number prevented**
2005	1.17 [1.12, 1.22]	-105	-105	1.20 [1.16, 1.25]	-79	-79
2006	1.53 [1.46, 1.59]	-318	-423	1.45 [1.40, 1.51]	-180	-259
2007	1.16 [1.11, 1.21]	-98	-521	1.09 [1.05, 1.13]	-36	-295
2008	0.97 [0.93, 1.01]	20	-501	1.19 [1.14, 1.23]	-75	-370
2009	0.87 [0.83, 0.91]	80	-421	0.95 [0.91, 0.98]	22	-348
2010	0.78 [0.75, 0.82]	135	-286	0.96 [0.93, 1.00]	14	-334
2011	0.79 [0.76, 0.83]	129	-157	0.93 [0.90, 0.97]	28	-306
2012	0.72 [0.69, 0.75]	175	18	0.83 [0.80, 0.86]	71	-235
2013	0.69 [0.66, 0.72]	196	214	0.77 [0.74, 0.79]	101	-134
2014	0.71 [0.68, 0.74]	164	378	0.73 [0.70, 0.76]	103	-31
2015	0.65 [0.63, 0.68]	202	580	0.81 [0.78, 0.84]	73	42
2016	0.65 [0.62, 0.68]	210	790	0.83 [0.80, 0.86]	69	111

FIT faecal immunochemical test, *CI* (bootstrap-estimated) confidence interval.

[a]2005 was the year of introduction of the screening programme. 2006 was the first full year of screening. The annual incidence rates that would be expected in 2005-2016 in the absence of screening were estimated by analysing the observed annual rates in 1997-2016 with an age-period-cohort model for men and an age-period model for women, i.e., the models providing the best fit to the observed rates. In both models, the values of parameters of the non-linear period effect were set to zero. All rates were age-standardised using the European standard population.
