# Supplementary material for: How a faecal immunochemical test screening programme changes annual colorectal cancer incidence rates: an Italian intention-to-screen study
Source: Br J Cancer. 2022 Apr 20;127(3):541–8. doi: 10.1038/s41416-022-01813-7 (PMC9345854; doi:10.1038/s41416-022-01813-7)
Supplement: Supplementary file 2 — Authorship agreement [file 41416_2022_1813_MOESM2_ESM.pdf]

Il giorno ven 25 mar 2022 alle ore 13:02 Mancini, Silvia <silvia.mancini@irst.emr.it> ha scritto:

Dear all,
I have updated the authorship of the paper submitted to the British Journal of Cancer "How a faecal immunochemical test screening programme changes annual colorectal cancer incidence rates: an Italian intention-to-screen study", with the following:

Lauro Bucchi[1], Silvia Mancini[1,*], Flavia Baldacchini[1], Alessandra Ravaioli[1], Orietta Giuliani[1], Rosa Vattiato[1], Federica Zamagni[1], Paolo Giorgi Rossi[2], Cinzia Campari[3], Debora Canuti[4], Enza Di Felice[5], Priscilla Sassoli de Bianchi[5], Stefano Ferretti[6], Nicoletta Bertozzi[5], Annibale Biggeri[7], Fabio Falcini[1,8] and the Emilia-Romagna Region Workgroup for Colorectal Screening Evaluation[§]

[1]Romagna Cancer Registry, Romagna Cancer Institute, *IRCCS Istituto Romagnolo per lo Studio dei Tumori (IRST) "Dino Amadori"*, Meldola, Forlì, Italy;
[2]Epidemiology Unit, Azienda Unità Sanitaria Locale – IRCCS di Reggio Emilia, Reggio Emilia, Italy;
[3]Cancer Screening Unit, Azienda Unità Sanitaria Locale – IRCCS di Reggio Emilia, Reggio Emilia, Italy;
[4]Cancer Screening Unit, Local Health Authority, Rimini, Italy;
[5]Department of Health, Regional Administration, Emilia-Romagna Region, Bologna, Italy;
[6]University of Ferrara and Local Health Authority, Ferrara, Italy;
[7]Unit of Biostatistics, Epidemiology and Public Health, Department of Cardiac, Thoracic, Vascular Sciences and Public Health, University of Padua, Padua, Italy;
[8]Cancer Prevention Unit, Local Health Authority, Forlì, Italy;
[§]A list of authors and their affiliations appears at the end of the paper.

Could you please confirm that you agree to these changes?

Thanks a lot,
Silvia Mancini

---------- Forwarded message ---------
Da: **Ravaioli, Alessandra** <alessandra.ravaioli@irst.emr.it>
Date: ven 25 mar 2022 alle ore 13:05
Subject: Re: BJC-A3338666R2 Initial Quality Check of your BJC Manuscript
To: Mancini, Silvia <silvia.mancini@irst.emr.it>

Yes, I agree
Thanks
Alessandra Ravaioli

---------- Forwarded message ---------
Da: **Giuliani, Orietta** <orietta.giuliani@irst.emr.it>
Date: ven 25 mar 2022 alle ore 13:07
Subject: Re: BJC-A3338666R2 Initial Quality Check of your BJC Manuscript
To: Mancini, Silvia <silvia.mancini@irst.emr.it>

I agree, thanks.
Orietta Giuliani
--
Orietta Giuliani
Dirigente Area Tecnostruttura

SC EPIDEMIOLOGIA CLINICA E SPERIMENTALE E REGISTRO TUMORI DELL'EMILIA-ROMAGNA
Unità Funzionale della Romagna
Tel. +39 0543 739452
orietta.giuliani@irst.emr.it
rtromagna@irst.emr.it
https://orcid.org/0000-0002-1843-3475

---------- Forwarded message ---------
Da: **Baldacchini, Flavia** <flavia.baldacchini@irst.emr.it>
Date: ven 25 mar 2022 alle ore 13:18
Subject: Re: BJC-A3338666R2 Initial Quality Check of your BJC Manuscript
To: Mancini, Silvia <silvia.mancini@irst.emr.it>

I agree, thanks.

Flavia Baldacchini
Statistico

U.O. Epidemiologia e Registro Tumori dell'Emilia Romagna - Unità Funzionale della Romagna
IRCCS Istituto Romagnolo per lo Studio dei Tumori (IRST) "Dino Amadori", Meldola (FC)
Tel. +39 0543 739452 - Fax. +39 0543 739459
flavia.baldacchini@irst.emr.it
rtromagna@irst.emr.it

---------- Forwarded message ---------
Da: **Zamagni, Federica** <federica.zamagni@irst.emr.it>
Date: ven 25 mar 2022 alle ore 13:23
Subject: Re: BJC-A3338666R2 Initial Quality Check of your BJC Manuscript
To: Mancini, Silvia <silvia.mancini@irst.emr.it>

I agree, thanks.

Federica Zamagni
Statistico

Registro Tumori della Regione Emilia-Romagna, Unità funzionale della Romagna
IRCCS Istituto Romagnolo per lo Studio dei Tumori (IRST) "Dino Amadori", Meldola, Forlì, Italy
Tel. +39 0543 739452 - Fax. +39 0543 739459
federica.zamagni@irst.emr.it
rtromagna@irst.emr.it
https://orcid.org/0000-0002-6129-6656

---------- Forwarded message ---------
Da: **Vattiato, Rosa** <rosa.vattiato@irst.emr.it>
Date: ven 25 mar 2022 alle ore 13:25
Subject: Re: BJC-A3338666R2 Initial Quality Check of your BJC Manuscript
To: Mancini, Silvia <silvia.mancini@irst.emr.it>

yes thanks
Rosa Vattiato

Rosa Vattiato

Dirigente Area Tecnostruttura

U.O. Epidemiologia e Registro Tumori dell'Emilia Romagna
Unità Funzionale della Romagna
IRCCS "Dino Amadori" - IRST S.r.l
Via Piero Maroncelli, 40 - 47014 Meldola (FC), Italy
Tel. +39 0543 739942 - Fax. +39 0543 739459
rosa.vattiato@irst.emr.it
rtromagna@irst.emr.it
https://orcid.org/0000-0002-3283-7572

---------- Forwarded message ---------
Da: **Falcini, Fabio** <fabio.falcini@irst.emr.it>
Date: ven 25 mar 2022 alle ore 13:38
Subject: Re: BJC-A3338666R2 Initial Quality Check of your BJC Manuscript
To: Mancini, Silvia <silvia.mancini@irst.emr.it>

yes thanks
Fabio Falcini

---------- Forwarded message ---------
Da: **Annibale Biggeri** <annibale.biggeri@ubep.unipd.it>
Date: ven 25 mar 2022 alle ore 13:45
Subject: R: BJC-A3338666R2 Initial Quality Check of your BJC Manuscript
To: Mancini, Silvia <silvia.mancini@irst.emr.it>

Perfect ! thanks

a.

---------- Forwarded message ---------
Da: **Giorgi Rossi Paolo** <Paolo.GiorgiRossi@ausl.re.it>
Date: ven 25 mar 2022 alle ore 13:48
Subject: R: BJC-A3338666R2 Initial Quality Check of your BJC Manuscript
To: Mancini, Silvia <silvia.mancini@irst.emr.it>

I agree, thanks.
Paolo

---------- Forwarded message ---------
Da: **Campari Cinzia** <Cinzia.Campari@ausl.re.it>
Date: ven 25 mar 2022 alle ore 13:48
Subject: R: BJC-A3338666R2 Initial Quality Check of your BJC Manuscript
To: Mancini, Silvia <silvia.mancini@irst.emr.it>

Yes, thanks.

Cinzia Campari
Centro Screening - AUSL di Reggio Emilia

---------- Forwarded message ---------
Da: **Bertozzi Nicoletta** <nicoletta.bertozzi@auslromagna.it>
Date: ven 25 mar 2022 alle ore 13:55
Subject: Re: BJC-A3338666R2 Initial Quality Check of your BJC Manuscript
To: Mancini, Silvia <silvia.mancini@irst.emr.it>

yes thanks
Nicoletta Bertozzi

---------- Forwarded message ---------
Da: **Di Felice Enza** < enza.difelice@regione.emilia-romagna.it>
Date: ven 25 mar 2022 alle ore 13:59
Subject: Re: BJC-A3338666R2 Initial Quality Check of your BJC Manuscript
To: Mancini, Silvia <silvia.mancini@irst.emr.it>

I agree, thanks
Enza

---------- Forwarded message ---------
Da: **Bucchi, Lauro** <lauro.bucchi@irst.emr.it>
Date: ven 25 mar 2022 alle ore 14:18
Subject: Re: BJC-A3338666R2 Initial Quality Check of your BJC Manuscript
25 March 2022
To: Mancini, Silvia <silvia.mancini@irst.emr.it>

I agree.

Lauro Bucchi

---------- Forwarded message ---------
Da: **Ferretti Stefano** <stefano.ferretti@unife.it>
Date: ven 25 mar 2022 alle ore 14:18
Subject: Re: BJC-A3338666R2 Initial Quality Check of your BJC Manuscript 25 March 2022
To: Mancini, Silvia <silvia.mancini@irst.emr.it>

I agree, thanks.

Stefano.

Prof. Stefano Ferretti
orcid.org/0000-0001-5241-2101
tel. 0532.455513, fax 0532.235375, cell 333.9264482
Skype: stefano.ferretti.fe; e-mail: frs@unife.it

- Dip. Medicina Traslazionale e per la Romagna, Università di Ferrara

- Registro Tumori della Regione Emilia-Romagna, Unità funzionale Azienda USL Ferrara

---------- Forwarded message ---------
Da: **Sassoli De Bianchi Priscilla** <PSassoli@regione.emilia-romagna.it>
Date: ven 25 mar 2022 alle ore 14:31
Subject: Re: BJC-A3338666R2 Initial Quality Check of your BJC Manuscript 25 March 2022
To: Mancini, Silvia <silvia.mancini@irst.emr.it>

I agree.

Priscilla Sassoli de Bianchi
Servizio Prevenzione collettiva e Sanità pubblica
Direzione Generale Cura della Persona Salute e Welfare
Regione Emilia-Romagna
Viale Aldo Moro 21 40127 Bologna
Tel. 051/ 527 -7041
Fax 051/ 527-7065

---------- Forwarded message ---------
Da: **Canuti Debora** <Debora.Canuti@Regione.Emilia-Romagna.it>
Date: ven 25 mar 2022 alle ore 14:38
Subject: Re: BJC-A3338666R2 Initial Quality Check of your BJC Manuscript 25 March 2022
To: Mancini, Silvia <silvia.mancini@irst.emr.it>

I agree, thanks.

---------- Forwarded message ---------
Da: **Registro Tumori** <rtromagna@irst.emr.it>
Date: ven 25 mar 2022 alle ore 14:41
Subject: Re: BJC-A3338666R2 Initial Quality Check of your BJC Manuscript
25 March 2022
To: Mancini, Silvia <silvia.mancini@irst.emr.it>

We agree thanks.
