# Supplementary material for: How a faecal immunochemical test screening programme changes annual colorectal cancer incidence rates: an Italian intention-to-screen study
Source: Br J Cancer. 2022 Apr 20;127(3):541–8. doi: 10.1038/s41416-022-01813-7 (PMC9345854; doi:10.1038/s41416-022-01813-7)
Supplement: Supplementary file 4 — Consortium authorship status [file 41416_2022_1813_MOESM4_ESM.pdf]

25 March 2022

RE:
BJC-A3338666R2
How a faecal immunochemical test screening programme changes annual colorectal cancer incidence rates: an Italian intention-to-screen study
by Bucchi L. et al.

I confirm that the consortium Emilia-Romagna Region Workgroup for Colorectal Screening Evaluation (rtromagna@irst.emr.it) qualifies for authorship status on this paper.

Thank you for your attention. I look forward to hearing from you.

Best regards,
Silvia Mancini*

*Corresponding author at the Romagna Cancer Registry, Romagna Cancer Institute (IRCCS Istituto Romagnolo per lo Studio dei Tumori (IRST) "Dino Amadori"), Meldola, Forlì, Italy.
E-mail: silvia.mancini@irst.emr.it
